# Autophagy Is Associated with Pathogenesis of *Haemophilus parasuis*

**DOI:** 10.3389/fmicb.2016.01423

**Published:** 2016-09-20

**Authors:** Yaning Zhang, Yufeng Li, Wentao Yuan, Yuting Xia, Yijuan Shen

**Affiliations:** Key Laboratory of Bacteriology, Ministry of Agriculture, College of Veterinary Medicine, Nanjing Agricultural UniversityNanjing, China

**Keywords:** *Haemophilus parasuis*, adenovirus, autophagy, pathogenesis, invasion

## Abstract

*Haemophilus parasuis* (*H. parasuis*) is a common commensal Gram-negative extracellular bacterium in the upper respiratory tract of swine, which can cause Glässer's disease in stress conditions. Research on the pathogenicity of *H. parasuis* has mainly focused on immune evasion and bacterial virulence factors, while few studies have examined the interactions of *H. parasuis* and its host. Autophagy is associated with the replication and proliferation of many pathogenic bacteria, but whether it plays a role during infection by *H. parasuis* is unknown. In this study, an adenovirus construct expressing GFP, RFP, and LC3 was used to infect *H. parasuis*. Western blotting, laser confocal microscopy, and electron microscopy showed that Hps5 infection induced obvious autophagy in PK-15 cells. In cells infected with strains of *H. parasuis* differing in invasiveness, the levels of autophagy were positively correlated with the presence of alive bacteria in PK-15 cells. In addition, autophagy inhibited the invasion of Hps5 in PK-15 cells. Autophagy related genes Beclin, Atg5 and Atg7 were silenced with RNA interference, the results showed that autophagy induced by *H. parasuis* infection is a classical pathway. Our observations demonstrate that *H. parasuis* can induce autophagy and that the levels of autophagy are associated with the presence of alive bacteria in cells, which opened novel avenues to further our understanding of *H. parasuis*-host interplay and pathogenesis.

## Importance

*Haemophilus parasuis* (*H. parasuis*) is a common commensal bacterium in the upper respiratory tract of swine, which causes Glässer's disease in stress conditions. But its pathogenicity was poorly understood. Autophagy, a crucial homeostasis mechanism, is associated with innate immunity and inflammatory response. Early studies mainly focused on the roles autophagy played in intracellular bacteria and viruses. We found that extracellular bacterium *H. parasuis* induced significant autophagy in PK-15 cells, especially, autophagy is associated with the presence of alive bacteria in cells, and which will help to inhibit the infection of *H. parasuis* in PK-15 cells.

## Introduction

*Haemophilus parasuis (H. parasuis)* is the etiological agent of Glässer's disease in swine, a systemic disease presenting as porcine polyserositis, polyarthritis and meningitis (Oliveira and Pijoan, 2004; Costa-Hurtado et al., 2013). Glässer's disease causes significant economic losses to the swine industry each year throughout the world (Morozumi et al., 1981). *H. parasuis* strains have been classified into 15 standard serotypes according to agar-gel-precipitation tests (AGPT) (Morozumi and Nicolet, 1986). Strains of *H. parasuis* are heterogeneous and differ in virulence (Kielstein and Rapp-Gabrielson, 1992). Serovars 1, 5, 10, 12, 13, and 14 are considered highly virulent and cause high mortality in pigs. Serovars 2, 4, 8, and 15 are less virulent, and 3, 6, 7, 9, and 11 are classified as avirulent (Kielstein and Rapp-Gabrielson, 1992; Nielsen, 1993). Although each *H. parasuis* serotype is associated with a characteristic level of virulence, the specific features that contribute to pathogenesis remain elusive.

Bacterial pathogenesis is a complex process, requiring multiple steps to establish infection and cause disease. Infection by *H. parasuis* requires invasion of host cells and evasion of host barriers such as serum complement (Cerda-Cuellar and Aragon, 2008) and macrophages (Yuan et al., 2012), thus resulting the inflammatory response. Virulent strains of *H. parasuis* can colonize and initiate infection by adhesion to and invasion of epithelial cells (Vanier et al., 2006; Frandoloso et al., 2012; Zhang et al., 2012, 2013), indicating that these processes are important first steps in infection. A previous report demonstrated that the ability of biofilm production of virulent strains *in vitro* is higher than avirulent strains isolated from nasal cavity, which is associated with colonization of *H. parasuis* (Jin et al., 2006). Adherence and invasion of virulent strains to cells play important roles in the infection of *H. parasuis*. Though the mechanism for virulent strains entering bloosdstream is poorly understood, the invasion of virulent strains to cells is associated with systemic infection (Olvera et al., 2009).

Intracellular bacterial pathogens have evolved many strategies to manipulate host cell functions during infection. For example, autophagy is a fundamental biological process, crucial in maintaining organismal homeostasis. It serves a housekeeping role by removing nonfunctioning proteins and damaged organelles, as well as intercellular pathogens, and has an adaptive role in protecting organisms against diverse pathogens (Cuervo, 2004; Levine and Kroemer, 2008). Autophagy can be induced by many kinds of cellular stresses, including hypoxia (Fang et al., 2015), nutrient (Steele et al., 2015) or growth factor deprivation (Ni et al., 2014), DNA damage (Houten et al., 2016), or pathogen infection (Steele et al., 2015; Winchell et al., 2015). Numerous studies have shown that autophagy plays an important role in the innate immune responses against infection (Yuan et al., 2012; Jo et al., 2013; Wang et al., 2015). Conversely, pathogens have evolved strategies to take advantage of autophagy for infection, replication and survival (Dorn et al., 2002; Campoy and Colombo, 2009; Castrejon-Jimenez et al., 2015).

Most researches confirmed that autophagy functioned as an effective mechanism to compete with intracellular bacteria. *H. parasuis* is able to adhere to and invade epithelial cells, but whether autophagy is associated with *H. parasuis* pathogenesis is unknown. In this study, western blotting, transmission electron microscopy, and confocal microscopy were used to detect and monitor autophagy in PK-15 cells infected by *H. parasuis*. We showed that autophagy induced by *H. parasuis* infection is associated with the invasiveness of *H. parasuis*. Our results have advanced our understanding of the interplay between autophagy and the pathogenesis of *H. parasuis*, which will be help to develop new drugs against *H. parssuis* infection.

## Materials and methods

### Reagents

Rabbit polyclonal antibody against LC3B was obtained from Sigma-Aldrich (Sigma, St. Louis, MO, USA), goat polyclonal antibodies against Beclin-1, Atg5, and Atg7, and GAPDH antibody were obtained from Santa Cruz Biotechnology (California, USA). Goat anti-mouse and anti-rabbit antibodies labeled with horseradish peroxidase (HRP) were acquired from Boster (Wuhan, China). The enhanced chemiluminescent (ECL) chromogenic kit was purchased from Biouniquer (Nanjing, China). Recombinant adenovirus tandom expressing GFP-RFP-LC3 was purchased from HANBIO (Shanghai, China), which was used to increase the expression of LC3 and facilitate the observation of fluorescence.

### Bacterial strains and growth conditions

Three standard serotypes of *H. parasuis* (serovars 4, 5, and 11; designated Hps4, Hps5, and Hps11) were used in this study. Bacteria were cultured at 37°C on Trypticase Soy Agar and in Trypticase Soy Broth (TSA and TSB, respectively; OXOID, Hampshire, England) supplemented with 0.01% nicotinamide adenine dinucleotide (NAD, Nanjing, JS, China) and 5% (v/v) inactivated bovine serum.

Bacteria were grown overnight and then inoculated in fresh TSB medium (1:100). Optical density at 600 nm was measured at 1 h intervals to establish growth curves. Bacteria from exponential phase cultures were serially diluted and plated onto TSA to determine the relationship between OD and viable bacterial counts (colony forming units).

### Cell culture

Porcine kidney (PK-15) cells were cultured at 37°C in a humidified 5% CO_2_ incubator and maintained in Dulbecco's Modified Eagle Media (DMEM, Gibco), supplemented with 10% heat-inactivated fetal bovine serum (FBS, Gibco) and antibiotics (100 U/ml of penicillin G and 100 mg/ml of streptomycin, Sigma, USA). Cells were sub-cultured when 90% cell confluence was attained. In 24-well tissue culture plates, cells were diluted in culture medium to a concentration of 10^5^ cells per well and incubated to 90% confluence.

### Infection experiments

Bacteria were grown overnight in TSB broth at 37°C with shaking as previously described (Cue et al., 1998), diluted 1:100 in fresh medium and incubated until reaching an optical density of 0.7 at 600 nm. Bacteria were pelleted by centrifugation at 8000 g for 1min, washed 3 times with PBS, and resuspended in DMEM at 0.25 OD (0.1OD = 1 × 10^8^ cells/ml) for the infection experiments (Yuan et al., 2012). PK-15 cells cultivated overnight in serum-containing medium were washed with sterile PBS and transferred to serum-free and antibiotic-free medium. The cells were infected with *H. parasuis* suspensions at different multiplicities of infection (MOI). At various time points post-infection, cells were treated with different methods as follows.

### Adherence and invasion assays

Adherence and invasion assays were performed as previously described (Cue et al., 1998; Vanier et al., 2006; Zhang et al., 2013) with modifications. Confluent monolayers of PK-15 cells were grown in 24-well plates and infected with *H. parasuis* suspensions at an MOI of 100. The plates were centrifuged at 800 × g for 10 min to promote contact of *H. parasuis* with the cell monolayer surface, incubated for 1 h in 5% CO_2_ at 37°C to allow bacterial adherence, and then vigorously washed five times with PBS to remove non-specifically attached bacteria.

For the adherence assay, after the removal of non-specifically attached bacteria, the plates were incubated for 10 min at 37°C with 100 μl of 0.025% trypsin. Following incubation, cells removed by scraping were transferred to sterile centrifuge tubes and centrifuged for 1 min at 12,000 rpm. The supernatant was removed and the pellet was resuspended with an equivalent volume of TSB. Serial dilutions of the cell lysate (100 μl) were plated onto Trypticase Soy Agar and incubated for 48 h at 37°C.

For the invasion assay, after the removal of non-specifically attached bacteria, culture medium containing two antibiotics (100 mg of penicillin G/ml and 5 mg of gentamicin/ml, Sigma) was added to each well, and the plates were incubated for 1 h at 37°C in 5% CO_2_ to kill any remaining extracellular *H. parasuis*. The monolayers were washed three times and cells were harvested as described above.

Bacterial adhesion/invasion was evaluated as the average number of bacteria per well (CFU, colony forming unit). All assays were performed in triplicate and repeated three times.

### Western blotting

Cell samples were lysed in RIPA buffer (Cell signaling Technologies) on ice for 30 min, then supernatant was collected through centrifugation at 12,000 rpm for 5 min. The lysate mixed with loading buffer was incubated for 8 min in a boiling water bath. Sample was loaded onto a 10% SDS-polyacrylamide mini-gel and subjected to electrophoresis. The proteins were transferred onto NC membranes (BioTrace^TM^ NT, Pall Corporation, United States) and blocked for 2 h at room temperature using 10% non-fat milk blocking buffer. Membranes were incubated overnight at 4°C with primary antibodies diluted to appropriate concentration in accordance with the instruction with PBST. After three rinses with PBST, the membranes were incubated for 45 min at room temperature with horseradish peroxidase-conjugated secondary antibody diluted 1:10000 (Wu et al., 2002). Bound antibody was visualized using an enhanced chemiluminescence detection kit (Super Signal West Pico; Pierce). For quantification, protein band density was analyzed using the image processing program ImageJ (Schneider et al., 2012).

### Confocal laser scanning microscopy

PK-15 cells were grown on coverslips in a 24-well plate and infected with adenovirus at an MOI of 1. After 24 h, a subset of the cells were pretreated with the autophagy activator rapamycin for 12 h, or the autophagy inhibitor 3-MA for 3 h, as positive and negative controls, respectively. Adenovirus infected/non-treated cells and adenovirus infected/3-MA treated were infected with *H. parasuis* at 0.1 MOI. Cells were washed five times with sterile PBS to remove non-specifically attached bacteria. Coverslips were mounted on microscope slides and samples were examined using a Zeiss LSM 700 confocal microscope.

### Transmission electron microscopy (TEM)

Cells in 6-well plates were infected with adenovirus at an MOI of 1 for 24 h and treated with rapamycin, 3-MA+ *H. parasuis, H. parasuis* or left untreated, respectively. Cells were harvested, centrifuged for 1 min at 300 × g, resuspended, and fixed in 2.5% glutaraldehyde at 4°C for 12 h, post-fixed in 1% osmium tetroxide, and finally dehydrated in a graded series of ethanol concentrations. Samples were treated in propylene oxide and embedded in epoxy resin for thin sectioning. Sections were double stained with uranyl acetate and lead citrate before being observed by TEM (Faulkner and Garduno, 2002; Zhang et al., 2015).

### Cell transfection

siRNA fragments specifically targeting Atg5, Atg7, Beclin-1, and one nonspecific random fragment (NC) were synthesized by Invitrogen. All siRNA sequences are listed in Table 1. For optimizing the conditions for gene silencing, PK-15 cells were seeded in 24-well plates at 1 × 10^5^ cells per well. At approximately 70% confluence, cells were incubated with several concentrations of siRNA fragments for different lengths of time, following the manufacturer's instructions. 24 h after transfection, cells were harvested and lysed for Western blot analysis. Then, the cells transfected with optimal siRNA fragments for Atg5/Atg7 (0.5 μg/well) and Beclin-1 (0.17 μg/well), were infected with *H. parasuis* suspensions at 0.1 MOI for 6 h, and then harvested and lysed for Western blot analysis.

**Table 1:** **siRNA sequences targeting ATG5/ATG7/Beclin1 gene**.

**Gene**	**Name**	**Sequence (5′–3′)**
ATG5	siRNA_21	GCUUCGAGAUGUGUGGUUUTT AAACCACACAUCUCGAAGCTT
	siRNA_62	CCCUCUAUCAGGAUGAGAUTT AUCUCAUCCUGAUAGAGGGTT
ATG7	siRNA_138	GGUAACUAUUGGUGUCUAUTT AUAGACACCAAUAGUUACCTT
	siRNA_846	GCAGCUCAUCGAAAGCCAUTT AUGGCUUUCGAUGAGCUGCTT
Beclin1	siRNA_101	CCUGGAUCGUGUUACCAUUTT AAUGGUAACACGAUCCAGGTT
	siRNA_125	GCUUACAGCUCCAUUACUUTT AAGUAAUGGAGCUGUAAGCTT

### Statistics

Three biological replicates were performed for every experiment and results were recorded as mean ± SD and GraphPad Prism 5 was used to analyze difference between groups (one-way ANOVA; Tukey's *post-hoc* test, ^***^p < 0.001, ^**^p < 0.01, ^*^p < 0.05). ImageJ software was used to confirm the band strength of WB and fold difference compared with control was labeled with number.

## Results

### *H. parasuis* infection induces autophagy

To determine whether infection by *H. parasuis* can induce autophagy, PK-15 cells were infected with adenovirus. After confirming successful expression of GFP-RFP-LC3, the PK-15 cells were infected with a highly virulent strain Hps5, in a dose- and time dependent manner (Figures S1, S2). We observed that Hps5 infection with a bacterial:cell multiplicity of infection (MOI) of 1:10 induced the most significant LC3- II level at 6 h (Figure S2, *p* < 0.001). Surprisingly, compared with control group, the level of LC3-II was decreased at 1–2 h and increased at 4–6 h during the infection (Figure S2).

**Figure 1:**
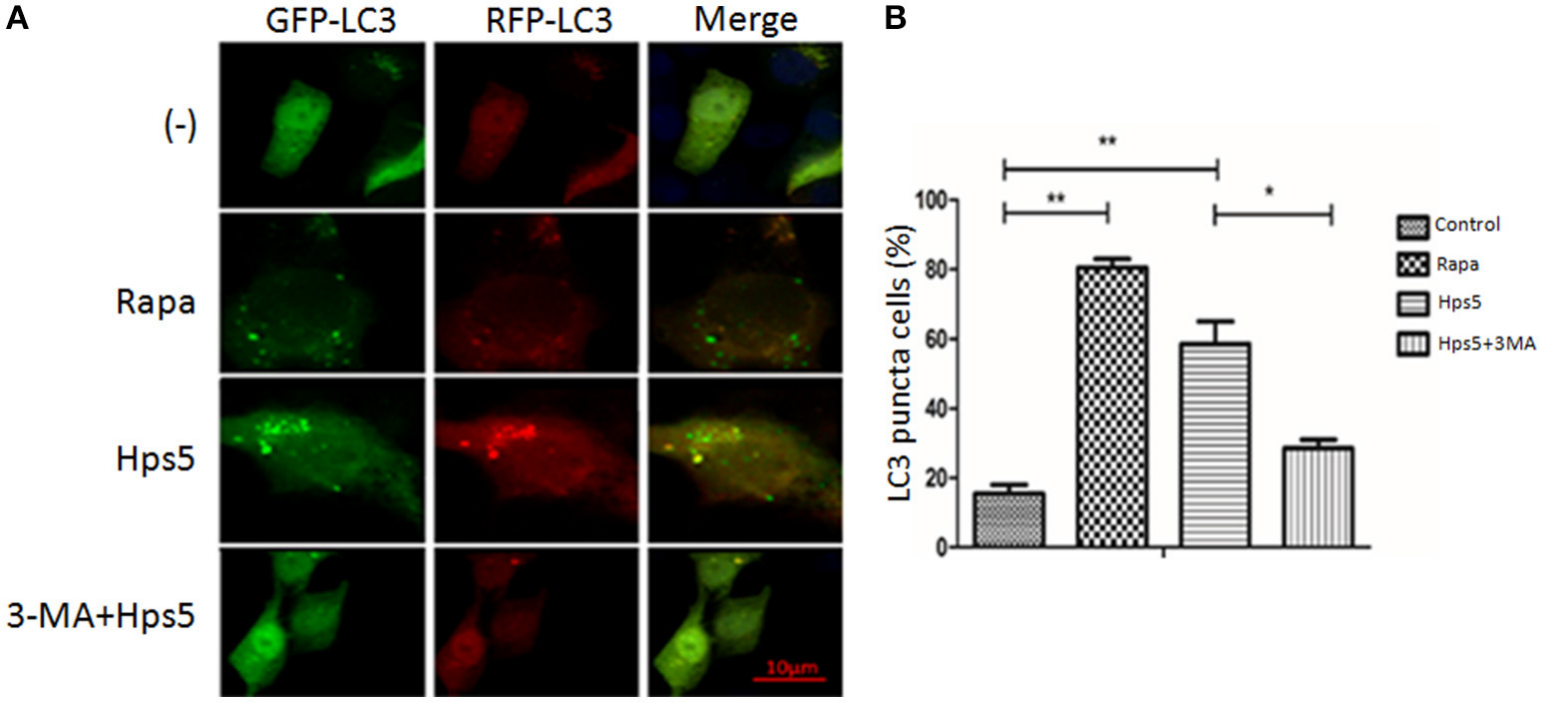
**Hps5 infection induces significant LC3 punctuation in PK-15 cells. (A)** PK-15 cells were infected with adenovirus expressing GFP-RFP-LC3 protein for 24 h. Then, cells were treated as follows. Control: Untreated cells. Hps5: Cells were super-infected with Hps5 for 6 h (MOI 0.1). Rapa: Cells were pre-treated with rapamycin (1 μM, 12 h) as positive control. 3-MA+Hps: Cells pretreated with 3-MA (3 mM, 3 h) and then infected with Hps5 for 6 h (MOI 0.1). **(B)** The LC3 puncta in each cell were counted and cells with more than 10 puncta were considered as positive. Values are from 100 cells per sample (one-way ANOVA; Tukey's *post-hoc* test, ^**^p < 0.01, ^*^p < 0.05). Data are representative of three individual experiments with similar results.

**Figure 2:**
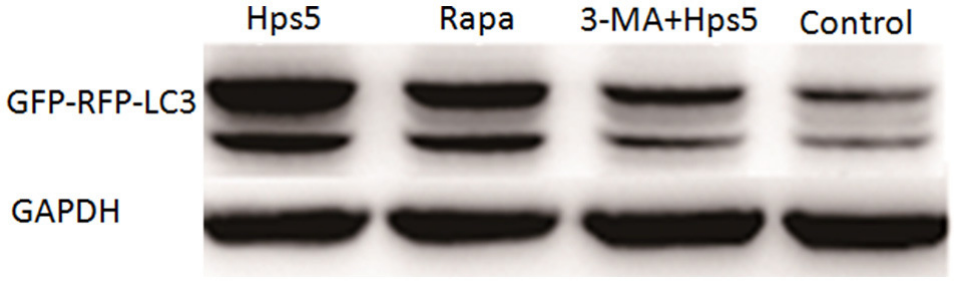
**The level of LC3 II in PK-15 cells infected by Hps5**. PK-15 cells were infected with adenovirus expressing GFP-RFP-LC3 for 24 h and then were infected with Hps5 for 6 h (MOI = 0.1). Before infection, cells were pretreated with either rapamycin (1 μM, 12 h) or 3-MA (3 mM, 3 h).

Autophagy induced in PK-15 cells was further confirmed with western blotting, confocal laser scanning and transmission electron microscopy. PK-15 cells were first infected with adenovirus, then cells were either (i) infected with Hps5, or (ii) treated with rapamycin for 12 h, or (iii) treated with 3-MA for 3 h before infected with Hps5, or (iv) left untreated and uninfected. As shown in Figures 1A,B, cells infected with Hps5 or treated with rapamycin exhibited increased LC3 punctuation. Consistent with these results, western blot analysis showed that LC3-II levels was increased significantly in cells infected with *H. parasuis* (Figure 2).

**Figure 3:**
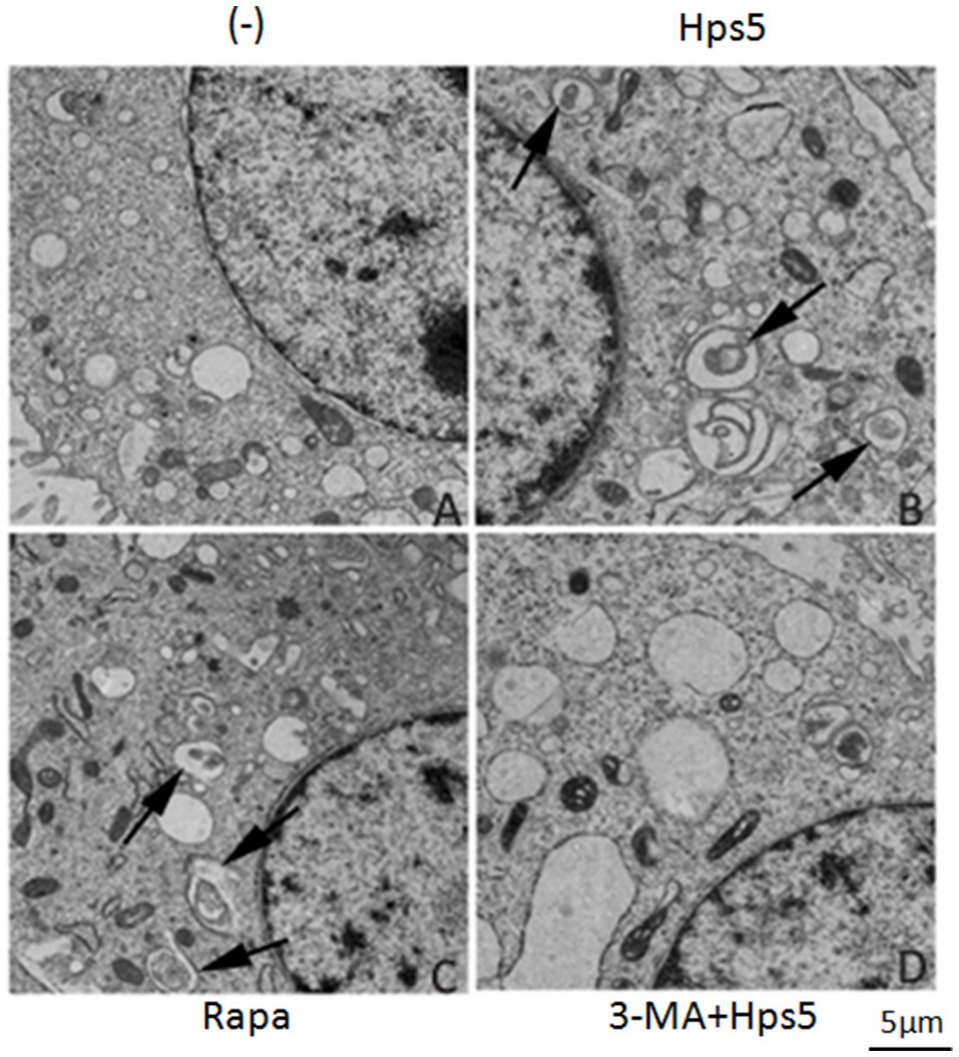
***H. parasuis* infection induced autophagosomes formation in PK-15 cells**. PK-15 cells infected with adenovirus expressing GFP-RFP-LC3 for 24 h were infected with Hps5 for 6 h (MOI = 0.1). Prior to infection, the cells were treated with rapamycin (1 μM, 12 h), 3-MA (3 mM, 3 h), or left untreated. After infection, cells were processed and examined by TEM. **(A)** Untreated cells (−). **(B)** Cells infected with Hps5. **(C)** Cells treated with rapamycin. **(D)** Cells treated with 3-MA and then infected with Hps5. Arrows indicate autophagosomes.

Transmission electron microscopy results showed that the autophagosome, a double membrane structure with heterogeneous contents, is apparent in cells infected by Hps5 (Figure 3B) or treated with rapamycin (Figure 3C). Uninfected/untreated cells (−) (Figure 3A) and cells treated with 3-MA before infection with *H. parasuis* (Figure 3D) have no obvious autophagosomes. In summary, these results demonstrate that Hps5 infection induces autophagy in PK-15 cells.

### Autophagy is associated with virulence of *H. parasuis*

**Figure 4:**
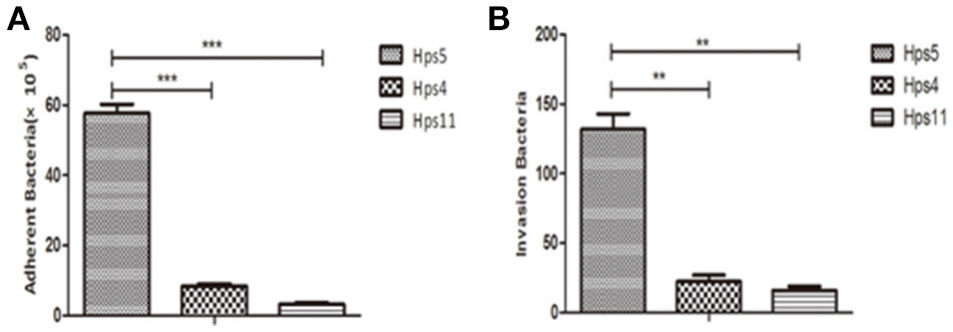
**Virulence associated with *H. parasuis* adherence and invasion to cells**. PK-15 cells were infected with Hps5 (highly virulent), Hps4 (less virulent), or Hps11 (avirulent) at 100 MOI, adherence **(A)** and invasion **(B)** results were recorded (one-way ANOVA; Tukey's *post-hoc* test, ^**^p < 0.01, ^***^p < 0.001).

Adherence and invasion assays were performed to determine if the autophagy induced by *H. parasuis* results from cell adherence or cell invasion. Briefly, PK-15 cells were infected at MOI 100 with strains Hps5 (highly virulent), Hps4 (less virulent), and Hps11 (avirulent) for 1 h. As shown in Figure 4A, Hps5 adhered to PK-15 cells was 6-fold more than Hps4 and 17-fold more than Hps11; In Figure 4B, invasion Hps5 in PK-15 cells was 5-fold more than Hps4 and 8-fold more than Hps11.

**Figure 5:**
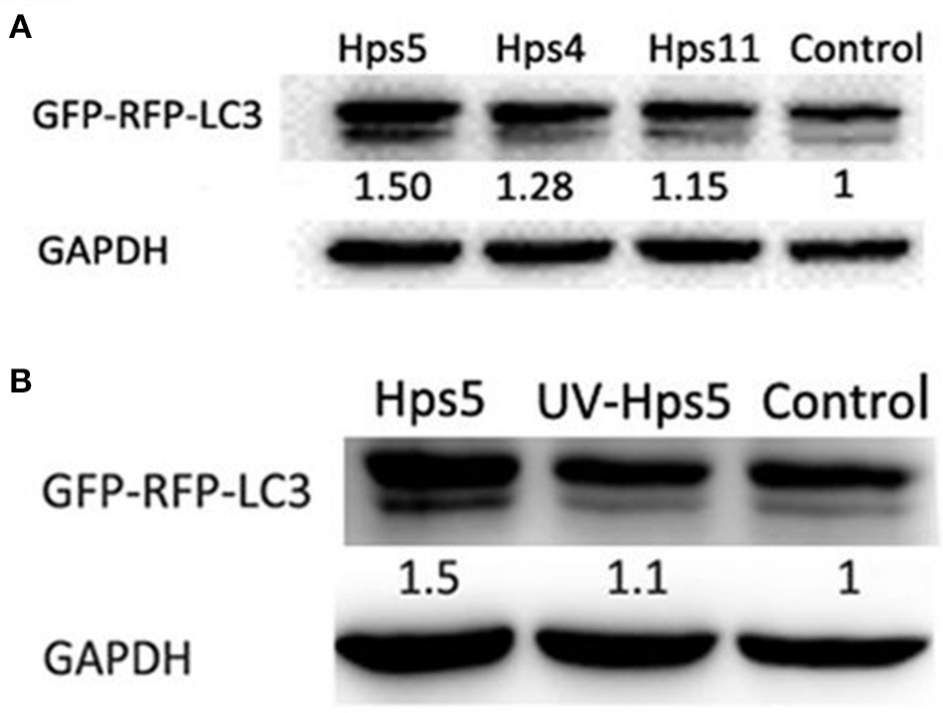
**Autophagy is associated with pathogenesis of *H. parasuis***. PK-15 cells infected with adenovirus expressing GFP-RFP-LC3 for 24 h were infected with Hps5 (highly virulent), Hps4 (less virulent), and Hps11 (avirulent) at 0.1 MOI **(A)** or infected with Hps5 and UV-inactivated Hps5 at 0.1 MOI **(B)**. The level of LC3 II was identified with western blotting and analyzed using the software ImageJ. GAPDH was used as a loading control. Data are representative of three individual experiments with similar results.

To explore the relationship between autophagy and *H. parasuis* pathogenesis, PK-15 cells preinfected with adenovirus at 0.1 MOI were infected with *H. parasuis* strains having different virulence: Hps5 (highly virulent), Hps4 (less virulent), and Hps11 (avirulent). Six hours after infection, cells were collected for western blot analysis. Using the mock-infected control as a baseline, the level ratio of LC3 II/GAPDH were calculated. The ratios were 1.50, 1.28, and 1.15 for the infection with Hps 5, Hps 4, and Hps 11, respectively (Figure 5A).

In another experiment, PK-15 cells pre-reated with adenovirus were infected with viable and UV-inactivated Hps5 at MOI 100, incubated, and then harvested for Western blot analysis to detect LC3-II level. The results showed that viable *H. parasuis* induces more autophagy than either the mock-infected control or inactivated Hps5 (Figure 5B). Given these results, we concluded that autophagy is predominately induced in porcine kidney epithelial cells by the presence of alive bacteria in cells.

### Autophagy inhibits *H. parasuis* invasion in PK-15 cells

**Figure 6:**
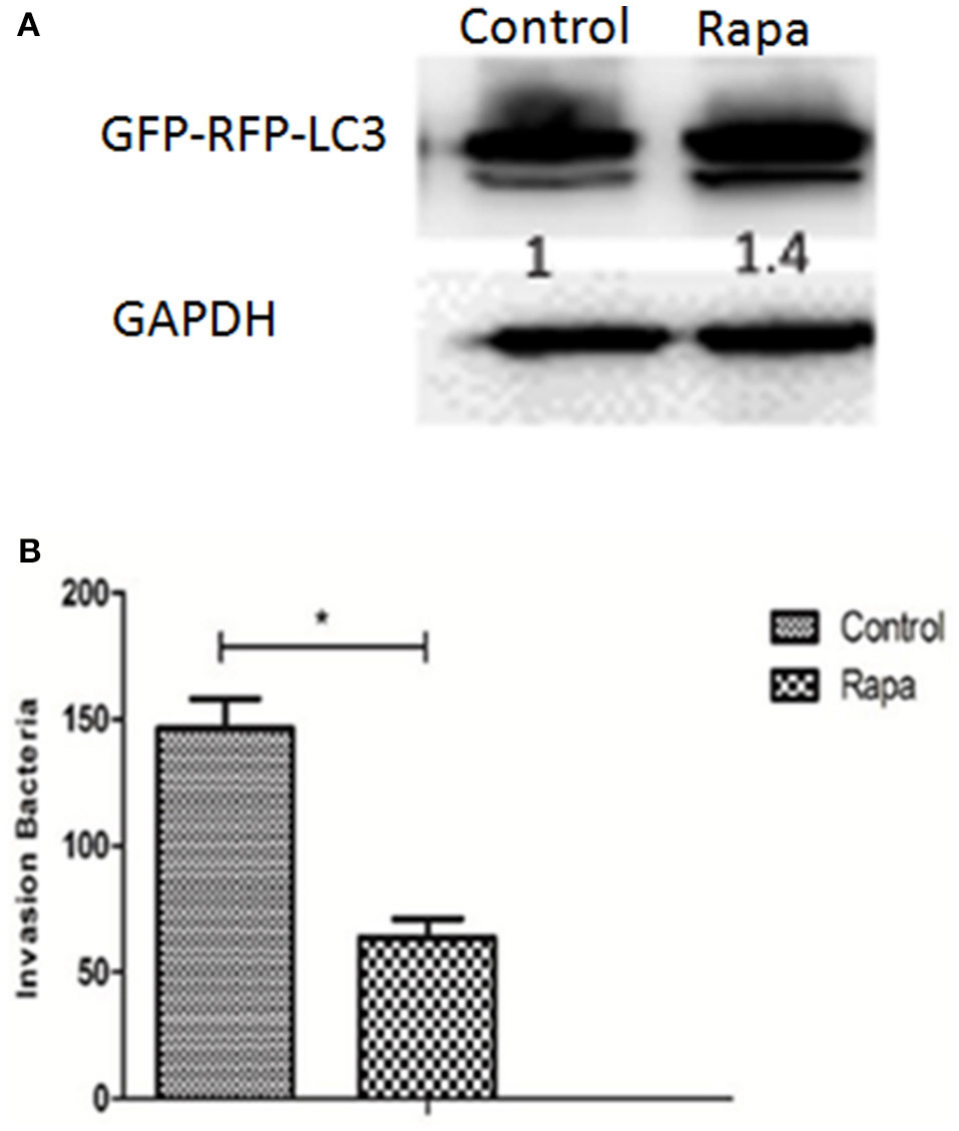
**Autophagy inhibits *H. parasuis* invasion to PK-15 cells. (A)** PK-15 cells were pretreated with adenovirus expressing GFP-RFP-LC3 for 24 h and then autophagy was induced in PK-15 cells by treated cells with rapamycin (1 μM, 12 h), the untreated cells were used as control. The level of LC3 II was identified with western blotting and analyzed using the software ImageJ. GAPDH was used as a loading control. **(B)** Invasion assay were performed using cells treated with rapamycin or not (one-way ANOVA; Tukey's *post-hoc* test, ^*^p < 0.05). Data are representative of three individual experiments with similar results.

Although autophagy ordinarily functions as a defense against bacterial invasion, some pathogens subvert the autophagic pathway to obtain nutrition for survival and replication, and ultimately to establish a persistent infection. In order to explore the role of autophagy in Hps5 infection, PK-15 cells were infected with adenovirus. After confirming successful expression of GFP-RFP-LC3, the cells were incubated with the autophagy activator rapamycin (1 μM, 12 h). Having confirmed that autophagy was induced in pretreated cells in comparison with untreated cells (Figure 6A), the treated and untreated cells were infected with Hps5 at MOI 100 and then the invasion assay was performed as described above. As shown in Figure 6B, the number of intracellular bacteria decreased significantly in rapamycin treated cells (*p* < 0.05). This result suggests that autophagy inhibited the invasion of *H. parasuis*.

### *H. parasuis* infection induces autophagy in PK-15 cells through the classical autophagy pathway

**Figure 7:**
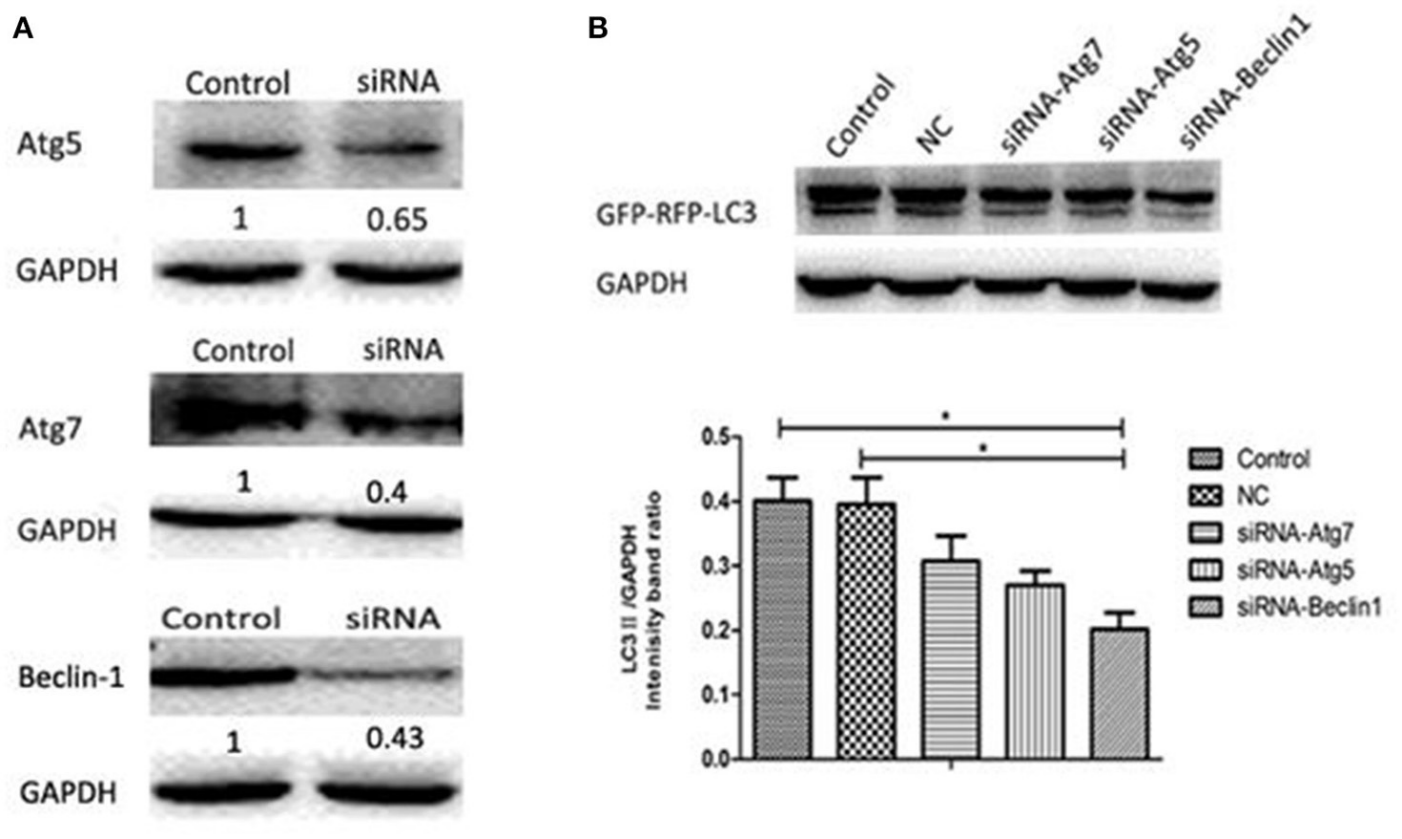
***H. parasuis* infection induced autophagy in PK-15 cells through the classical autophagy pathway. (A)** Western blotting of Beclin-1/ Atg7/Atg5. GAPDH was probed as a loading control in **(A,B)**. **(B)** PK-15 cells were pretreated with adenovirus expressing GFP-RFP-LC3 for 24 h and then transfected with siRNA-Beclin1, siRNA-Atg5, siRNA-Atg7 for 24 h. Then the cells were infected with Hps5 for 6 h. Before infection, the cells were also treated with negative control siRNA. The level of LC3 II was identified with western blotting and analyzed using the software ImageJ (one-way ANOVA; Tukey's *post-hoc* test, ^*^p < 0.05). Data are representative of three individual experiments with similar results.

The classical autophagy pathway is mediated by a large number of autophagy related genes (Atg), among which are Atg5, Atg7, and Beclin-1 (Atg6) (Ohsumi and Mizushima, 2004; Yuan et al., 2012). To determine whether this pathway is involved when *H. parasuis* infection induces autophagy, siRNA fragments specifically targeting Atg5 (siRNA-21/siRNA-42), Atg7 (siRNA-138/siRNA-846), Beclin-1 (siRNA-101/siRNA-125) (Table 1) and one nonspecific random fragment (NC) were synthesized and used in gene expression silencing experiments. The concentration and timing of interference effects were firstly determined by western blot analysis, the best interference result was shown in Figure 7A. After transfection using optimal conditions, PK-15 cells were infected by Hps5 (highly virulent), and untransfected cells were infected as controls. The level of LC3-II was significantly reduced by all three specific interfering fragments (Figure 7B). We conclude that Atg5, Atg7, and Beclin-1 play an important roles in the signaling pathway of the autophagy induced in PK-15 cells by *H. parasuis* infection.

## Discussion

Autophagy, a crucial homeostasis mechanism, is associated with innate immunity and the inflammatory response. In recent years, the roles of autophagy in intracellular bacterial infections have attracted increasing interest. Pathogenic microorganisms such as *Pseudomonas aeruginosa* (Yuan et al., 2012) and *Salmonella* (Birmingham and Brumell, 2006) activate autophagy in host cells, while others have evolved mechanisms to subvert this host cell defense mechanism and replicate successfully. For example, some pathogens escape into the cytoplasm to avoid lysosomal killing (Pizarro-Cerda and Cossart, 2006), others remain inside vacuolar phagosomes and hamper their maturation into lysosomes, and some utilize the host's defense mechanisms to their advantage (Colombo, 2005), such as *Brucella* (Brumell, 2012). Most studies of bacterial autophagy have focused on intracellular pathogens, such as *Mycobacterium tuberculosis* (Kimmey et al., 2015) and *Brucella*, but extracellular pathogens such as *Pseudomonas aeruginosa* (Yuan et al., 2012) and *Acinetobacter baumannii* (Rumbo et al., 2014) are also known to induce autophagy through cell invasion.

*H. parasuis*, a Gram-negative extracellular bacterium, causes significant losses on the swine industry throughout the world. Research on the pathogenicity of *H. parasuis* has focused mainly on immune evasion and bacterial virulence factors, while few studies have examined interactions between *H. parasuis* and its host, including the relationship between autophagy and *H. parasuis* infection. Previous studies have demonstrated that the ability of *H. parasuis* to invade PK-15 cells is correlated with virulence (Frandoloso et al., 2012). In this study we examined the relationship between autophagy and *H. parasuis* infection. We showed that *H. parasuis* can induce obvious autophagy in PK-15 cells at MOI of 0.1 (Figure S1). Interestingly, *H. parasuis* can decrease autophagy in the early phase (1–2 h) of *H. parasuis* infection, but which increased in 4–6 h after infection, which may suggest an evolve mechanism for the interplay between *H. parasuis* and host. Other pathogens are also known to depress autophagy; for example, the human cytomegalovirus (HCMV) inhibits autophagy early in infection by increasing the expression of the rapamycin target protein (Chaumorcel et al., 2008). We also demonstrated a positive correlation *between H. parasuis* invasion and the level of induced autophagy. Consequently, there is a need to further explore the factors that influence the invasive ability of *H. parasuis*.

The invasion ability of *H. parasuis* is positively correlated with the virulence (Frandoloso et al., 2012). In our study, different virulent strains were used to adhere to and invade PK-15 cells with different MOI, the results showed that the infection with 100 MOI of *H. parasuis* gived best adherence and invasion (data not shown). So, we can conclude that the ability of adherence and invasiveness of *H. parasuis* is positively correlated with the virulence of bacteria. In addition, we also found different virulent strains of *H. parasuis* induced different level of autophagy in PK-15 cells. The highly virulent Hps5 induced stronger autophagy level than less virulent Hps4 and avirulent Hps11. Compared with live Hps5 infection, UV-inactivation Hps5 induced weak autophagy. Combining these results, we can draw a conclusion that autophagy induced by *H. parasuis* is positively associated with the presence of alive bacteria in cells (invasion).

Pre-treat PK-15 cells with rapamycin, the invasion result showed that autophagy effectively inhibited the invasion of Hps5, which suggested that autophagy is a defense mechanism of PK-15 cells to inhibit the invasion of *H. parasuis*.

To date, more than 30 autophagy related genes (Atg) and their proteins have been identified. These proteins take an active part in the shaping of isolation membranes and autophagosomes, and participate in the fusion process of autophagosome and lysosome, constituting the core mechanisms of autophagy (Yamaguchi and Otsu, 2012). Among them, 15 genes (Atg1-10, Atg12-14, Atg16, Atg18) are essential for all types of autophagy (Longatti and Tooze, 2009). Atg7-Atg4-Atg8, Atg12-Atg7-Atg5 are two classical signaling pathways of autophagy, which are both dependent on Atg6 (Beclin-1 in mammals). In our study, three autophagy related genes Beclin-1, Atg5, and Atg7 were silenced with siRNA fragments. We found that proteins expressed by these three genes played important roles in the autophagy process induced by *H. parasuis*. Thus, our data identified the classical Beclin-1–Atg5–Atg7 autophagy pathway in PK-15 cells induced by *H. parasuis*.

In order to effectively invade PK-15 cells, *H. parasuis* decreased autophagy level at early stage of infection. With the increasing of alive bacteria in cells, high level of autophagy was induced to inhibit the invasion of *H. parasuis*. So, autophagy is a defense mechanism of PK-15 cells to inhibit the infection of *H. parasuis*. Our study is helpful to elucidate the signal pathways induced by *H. parasuis* infection and development of novel drugs against this bacteria.

## Author contributions

YZ, perform experiment and organize manuscript. YL, Design experiment and modify manuscript. WY, Figures making. YX, Bacteria isolation. YS, Bacteria culturing.

### Conflict of interest statement

The authors declare that the research was conducted in the absence of any commercial or financial relationships that could be construed as a potential conflict of interest.

## Supplementary material

Figure S1 **Optimization of MOI of *H. parasuis* infection associated with autophagy**. PK-15 cells pretreated with adenovirus expressing GFP-RFP-LC3 for 24 h were infected with Hps5 at different MOIs. Prior to infection, cells were treated with rapamycin (1 μM) for 12 h. The level of LC3 II was detected by western blotting **(A)** and quantitated using the ImageJ software package **(B)** (one-way ANOVA; Tukey's *post-hoc* test, ^**^*p* < 0.01, ^***^*P* < 0.001). GAPDH was used as a loading control.

Figure S2 **Optimization of time of *H. parasuis* infection associated with autophagy**. PK-15 cells pretreated with adenovirus expressing GFP-RFP-LC3 for 24 h were infected with Hps5 (MOI = 0.1) or mock infected and analyzed at 1, 2, 4, and 6 h post-infection. The level of LC3 II was detected by western blotting and quantitated using the ImageJ software package (one-way ANOVA; Tukey's *post-hoc* test, ^**^*p* < 0.01, ^***^*P* < 0.001).
