# Supplementary figures and images for: Autophagy Is Associated with Pathogenesis of Haemophilus parasuis
Source: Front Microbiol. 2016 Sep 20;7:1423. doi: 10.3389/fmicb.2016.01423 (PMC5028388; doi:10.3389/fmicb.2016.01423)

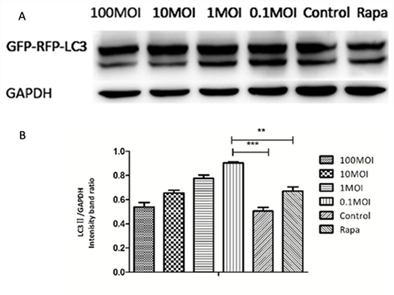

Supplement: Figure S1 — Optimization of MOI of H. parasuis infection associated with autophagy. PK-15 cells pretreated with adenovirus expressing GFP-RFP-LC3 for 24 h were infected with Hps5 at different MOIs. Prior to infection, cells were treated with rapamycin (1 μM) for 12 h. The level of LC3 II was detected by western blotting (A) and quantitated using the ImageJ software package (B) (one-way ANOVA; Tukey's post-hoc test, **p < 0.01, ***P < 0.001). GAPDH was used as a loading control. [file Image1.TIF]

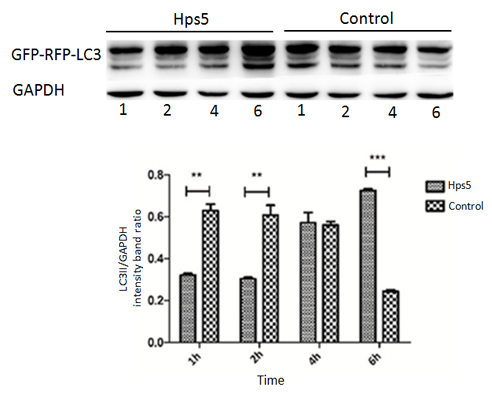

Supplement: Figure S2 — Optimization of time of H. parasuis infection associated with autophagy. PK-15 cells pretreated with adenovirus expressing GFP-RFP-LC3 for 24 h were infected with Hps5 (MOI = 0.1) or mock infected and analyzed at 1, 2, 4, and 6 h post-infection. The level of LC3 II was detected by western blotting and quantitated using the ImageJ software package (one-way ANOVA; Tukey's post-hoc test, **p < 0.01, ***P < 0.001). [file Image2.TIF]
